# An overview of the ameliorative efficacy of *Catharanthus roseus* extract against Cd^2+^ toxicity: implications for human health and remediation strategies

**DOI:** 10.3389/fpubh.2024.1327611

**Published:** 2024-03-08

**Authors:** Mohammad Hashim, Hussain Arif, Baby Tabassum, Shahnawaz Rehman, Priya Bajaj, Rekha Sirohi, Mohd Faizan Ali Khan

**Affiliations:** ^1^Department of Biochemistry, S. S. Faculty of Science, Mohammad Ali Jauhar University, Rampur, UP, India; ^2^Toxicology Laboratory, Department of Zoology, Govt. Raza P. G. College, Rampur, UP, India; ^3^Department of Biochemistry, Faculty of Life Sciences, Aligarh Muslim University, Aligarh, UP, India; ^4^IIRC-1, Department of Biosciences, Integral University, Lucknow, UP, India; ^5^Department of Zoology, Govt. P. G. College Noida, Noida, India; ^6^Environmental Engineering Laboratory, Department of Civil Engineering, Aligarh Muslim University, Aligarh, India

**Keywords:** cadmium, toxicity, exposure, human health, oxidative stress, *Catharanthus roseus*

## Abstract

Rapid industrialization has led to an increase in cadmium pollution, a dangerously toxic heavy metal. Cadmium (Cd) is released into the environment through industrial processes and can contaminate air, water, and soil. This pollution poses a significant risk to human health and has become a pressing concern in many industrialized areas. Due to its extended half-life, it leads to a range of health problems, including hepato-nephritic toxicity, brain damage, and degenerative bone disorders. Intoxication alters various intracellular parameters, leading to inflammation, tissue injury, and oxidative stress within cells, which disrupts normal cellular functions and can eventually result in cell death. It has also been linked to the development of bone diseases such as osteoporosis. These adverse effects highlight the urgent need to address cadmium pollution and find effective solutions to mitigate its impact on human health. This article highlights the Cd-induced risks and the role of *Catharanthus roseus* (*C. roseus*) extract as a source of alternative medicine in alleviating the symptoms. Numerous herbal remedies often contain certain bioactive substances, such as polyphenols and alkaloids, which have the power to mitigate these adverse effects by acting as antioxidants and lowering oxidative cell damage. Research conducted in the field of alternative medicine has revealed its enormous potential to meet demands that may be effectively used in safeguarding humans and their environment. The point of this review is to investigate whether *C. roseus* extract, known for its bioactive substances, is being investigated for its potential to mitigate the harmful effects of cadmium on health. Further investigation is needed to fully understand its effectiveness. Moreover, it is important to explore the potential environmental benefits of using *C. roseus* extract to reduce the negative effects of Cd. This review conducted in the field of alternative medicine has revealed its enormous potential to meet demands that could have significant implications for both human health and environmental sustainability.

## 1 Introduction

Heavy metals pose a major environmental concern due to their toxicity, inability to biodegrade, ability to accumulate biologically, and carcinogenic properties. These factors constitute a serious hazard to the environment and human health (1). In contrast to organic pollutants, heavy metals exhibit non-biodegradable characteristics and have a propensity to accumulate within vegetation, animals, and human beings subsequent to their discharge into the environment. This accumulation can have detrimental effects on the overall well-being of all organisms (1–3).

Exposure to heavy metals in the workplace and other settings causes both short-term and long-term health problems, making them a serious environmental concern. Epidemiological research has shown that exposure to low concentrations of harmful metals in the environment leads to the development of numerous disorders (4). Therefore, knowledge of the molecular processes, physiological adjustments, and physio pathological changes brought on by exposure to heavy metals is becoming increasingly important. The Agency for Toxic Substances and Disease Registry (ATSDR) ranks Cd among the top five most harmful environmental pollutants, as it is a non-essential metal (5). Many people, including governments and scientists, have been concerned about the toxicity of Cd ever since the Itai-itai sickness first appeared in Japan in 1912 (6). According to Wang et al. (7), industrial activity in many developing nations continues to pose a challenge to the reduction of mercury pollution, despite the fact that several countries have implemented various measures to that effect.

Moreover, Cd ion is a highly toxic heavy metal that is commonly found in the environment due to industrial activities; approximately 45,000 tons of Cd are released into the environment annually through various causes, including volcanic emissions, the combustion of fossil fuels, and the erosion of sedimentary materials. Such contamination affects landowners, lakes, and rivers, the quality of the air, and aquatic life (8). According to the United States Geological Survey Mineral Yearbook, which contains valuable insights into worldwide Cd output, the following countries supplied the most significant amounts between 2017 and 2020: Australia (29%), China (20%), Germany (19%), Peru (11%), and others (29%) (4). It can accumulate in various organs of the body, such as the liver and kidneys, causing various detrimental effects on human health. Understanding these diverse sources and regional variations is crucial for developing effective strategies to mitigate Cd exposure and protect public health. Therefore, it is crucial to investigate potential remedies that can mitigate the negative consequences of Cd2+ toxicity. The study of *C. roseus* extract is significant because it possesses bioactive compounds that have shown potential for combating heavy metal toxicity and its associated health problems.

In recent years, researchers and environmentalists have explored various avenues to mitigate the adverse effects of Cd toxicity. One promising avenue is the utilization of plant-based remediation strategies, where certain plant species exhibit the ability to accumulate and detoxify heavy metals from the soil. *Catharanthus roseus*, commonly known as Madagascar periwinkle or *Vinca rosea*, has gained attention for its potential in phytoremediation due to its unique ability to hyperaccumulate heavy metals, including Cd. Moreover, *C. roseus* is well-known for its rich repertoire of bioactive compounds (5), making it a subject of interest not only for environmental remediation but also for its potential implications in human health.

The bioactive compounds found in *C. roseus*, such as alkaloids, flavonoids, and terpenoids, have been reported to possess antioxidant and chelating properties (9, 10). These properties suggest that *C. roseus* extracts may play a pivotal role in ameliorating Cd-induced oxidative stress and cellular damage (5). Furthermore, the potential use of *C. roseus* as a dietary supplement holds promise for preventing or mitigating Cd toxicity in humans, thereby contributing to the development of novel strategies for human health protection (11, 12).

This review aims to provide a comprehensive overview of the existing literature on the ameliorative efficacy of *C. roseus* extract against Cd2+ toxicity. We will delve into the biochemical and molecular mechanisms underlying the protective effects of *C. roseus* against Cd-induced toxicity, emphasizing its role in mitigating oxidative stress, inflammation, and cellular damage. Additionally, we will explore the implications of *C. roseus* in human health, considering its potential as a dietary supplement to counteract Cd toxicity.

To provide a holistic perspective, this review will incorporate findings from both *in vitro* and *in vivo* studies, highlighting the diverse experimental approaches employed to evaluate the efficacy of *C. roseus* in mitigating Cd toxicity. Moreover, we aim to contribute valuable insights to the fields of environmental science, pharmacology, and human health. Through a synthesis of existing knowledge, this review seeks to stimulate further research and innovation in the development of sustainable remediation strategies and health interventions in the face of Cd contamination.

## 2 Adverse effects on a population caused by heavy metals

In recent years, there has been growing concern about the impact of environmental toxins, particularly heavy metals, on human health and the ecosystem. Rapid industrialization has led to widespread pollution, and heavy metals, such as cadmium (Cd), play a significant role in these environmental issues (13). According to the United Nations Environment Program/Global Program of Action (UNEP/GPA) (14), World Health Organization (WHO), and the European Parliamentary Council, heavy metals can originate from either natural or artificial sources. Agricultural and geological effluents, electronic waste, cosmetic waste, pharmaceutical waste, industrial waste, and domestic waste contribute to the contamination (15–17).

Epidemiological studies, as reported by Agency for Toxic Substances and Disease Registry (ATSDR) (5), indicate that over 5 million people are exposed to dangerous metals like Cd annually, known for their toxic properties and cumulative effects (18). Hashim et al. (17) note that toxicity from mercury (Hg) still affects 40,000–80,000 people worldwide, 200 million from lead (Pb), and approximately 250 million from Cd. Also, according to the WHO, 90% of the world population breathes polluted air, which is responsible for the deaths of approximately 7 million people each year (19). A 2020 research study by United Nations Children’s Fund (UNICEF) revealed that a majority of the 800 million children affected by lead worldwide reside in India. It is estimated that air pollution causes the death of approximately one out of eight people worldwide, and this rate is increasing in regions where population density is higher and thus air pollution is higher (20).

### 2.1 Economic consequences associated with Cd exposure

The global prevalence of trace metal problems, particularly Cd, continues to escalate significantly despite considerable financial investments in developing cutting-edge technologies and services to remove these contaminants from soils (21). This persistent issue is primarily attributed to inadequate awareness and economic limitations, especially in developing nations (22). Chronic exposure to Cd has been associated with a spectrum of health issues, including kidney dysfunction (5), bone disorders (18), and an increased risk of cancer (23). These health impacts impose significant economic burdens, as medical expenses and productivity losses contribute to the overall cost (5). The economic consequences extend beyond healthcare, impacting resources that could otherwise be directed toward development and improving living conditions.

The economic burden stemming from Cd exposure is substantial, with the costs associated with treating illnesses and rehabilitating affected individuals placing a strain on financial resources (5). Urgent action and stricter regulations are imperative to mitigate the detrimental effects of heavy metal exposure in both India and Europe.

Cadmium, one of the most common heavy metals, has a historical context as it was used in World War I as a tin substitute and in the paint industry as a pigment. It accumulates in the human body through various sources, primarily ores of zinc, copper, or lead. The extraction and processing of these ores release large amounts of Cd into the hydrosphere, atmosphere, soil, and food chain (24). As a result, Cd pollution has broader environmental consequences, affecting ecosystems and biodiversity. Industries reliant on natural resources, such as fisheries and forestry, experience declines in productivity, and revenue due to Cd-induced environmental degradation.

There are over 50 elements classified as heavy metals, with 17 considered particularly toxic. Cd stands out as abundant, pervasive, non-essential, and the most harmful divalent industrial and environmental heavy metal (24). Recognizing the widespread impact of Cd pollution on both human health and the environment, comprehensive strategies, including stricter regulations and effective remediation methods, are essential to address the economic repercussions associated with Cd exposure.

### 2.2 Toxicological mechanism associated with Cd exposure

Cadmium (Cd) is ubiquitously present in environmental matrices such as air, water, soil, and biological tissues, existing predominantly in bound forms rather than in its elemental state. Its classification as a Group 1 carcinogen by the International Agency for Research on Cancer underscores its profound implications for human health (25). Cd toxicity poses a formidable challenge due to the absence of a specific, safe, and efficacious detoxification protocol. Furthermore, its protracted elimination half-life and wide-ranging organ toxicity augment its hazardous nature, making it one of the most poisonous heavy metals in the environment. (17). Unsal et al. (26) found that Cd reduces the levels of total glutathione and protein-bound sulfhydryl groups in various cell lines. This reduction leads to an increase in reactive oxygen species (ROS) like hydroxyl radicals, hydrogen peroxide, and superoxide ions. Elevated ROS levels are associated with enhanced lipid metabolism, high lipid peroxidation, modification of cellular oxidation states, DNA breakage, changes in gene expression, or even cell death (17, 27–29). Although Cd does not undergo redox reactions like copper, it can induce DNA damage through the generation of ROS in the presence of hydrogen peroxide. Previous studies have demonstrated Cd′s ability to harm plasmid DNA by interacting with nitrogenous bases and enzymes involved in their function (30). Moreover, Cd exposure is known to cause oxidative stress, leading to DNA damage, emphasizing the importance of understanding the mechanisms through which Cd induces such harm for the development of effective protective strategies.

### 2.3 Cadmium toxicity and its detrimental impact on human health

Cadmium-induced itai-itai disease causes a variety of symptoms, including reduced bone mineralization, a high incidence of injuries, an elevated risk of osteoarthritis, and severe bone discomfort. In Japan, an explosion of the illness was reported (31, 32). Severe abdominal discomfort, nausea, vomiting, diarrhea, headache, and/or vertigo are all symptoms of acute Cd toxicity, which can result in death within 24 h or up to 2 weeks later due to liver and kidney damage. Bone illness, including osteoporosis and spontaneous bone fracture, is also associated with chronic Cd exposure, along with respiratory issues and renal dysfunction (18, 23). Other effects of Cd exposure include psychological issues, damage to the central nervous system, diarrhea, abdominal discomfort, severe vomiting, reproductive problems, infertility, and DNA damage (33, 34).

Cadmium is not an essential element, so it does not enter the body through a specific transport system (35). Instead, it can affect hormones and breathing systems in humans and invertebrates. Cd can also cause oxidative stress, which can lead to kidney damage and liver problems. This is because Cd can bind to estrogen receptors in cells and mimic the effects of estrogens. There is also some evidence to suggest that being exposed to Cd may increase one’s risk of developing prostate cancer (36, 37). However, some grazing animals are born with a low amount of Cd in their bodies, but it still builds up gradually. It has a known biological purpose, although it imitates the effects of similar divalent metals, which are necessary for a wide range of biological activities, according to Peana et al. (38). In addition to the testicles and ovaries, the liver and kidneys are the main tissues, which have multiple cytotoxic and metabolic effects because of Cd buildup or overdose. The liver accumulates most of the Cd that is easily collected. It also makes less Cd available to organ systems like the kidneys and testicles, which are more sensitive to its harmful effects (31, 39). Although most domesticated animals are born with a small concentration of Cd, the two organs listed above accumulate 40–80% of the body’s overburden of Cd over the course of a lifetime. Low-level exposures, like those found in the environment, cause the kidneys to store about half of the body’s Cd. The cortex has concentrations that are 1.25 times higher than the rest of the kidney, and the amount of Cd that is not linked to MTs is directly related to the amount of tubular damage (40).

The concentration of Cd in the livers of individuals who are not occupationally exposed grows steadily with age. Additionally, the concentration grows in the renal cortex until the age of 50–60 years, after which it levels out or even declines (23, 41, 42). In the human kidney, it usually builds up in the segment one section of the proximal tubule, which also causes phosphate reabsorption issues (Fanconi), amino acid, protein, and bicarbonate by damaging transport proteins and mitochondria, which may cause tubular cells to die (43–45). Cells contain almost 90% of the Cd in the blood. Blood Cd levels in persons who have never been exposed to the metal in the workplace are typically below 0.5 μg/100 mL. The amount of Cd in one’s blood during Cd exposure is a good predictor of the amount absorbed over the preceding months (41). People who have been exposed to a lot of Cd in the past, like retired workers, may have a blood concentration that is mostly affected by the amount of Cd in their bodies if the amount of Cd released from storage sites is higher than the amount that is being absorbed at the moment. Some research suggests that zinc and iron can mitigate Cd toxicity (4, 46, 47).

Cadmium-induced cell death is caused by the release of harmful molecules, the increase in calcium levels, a certain protein being turned on, a protective protein being turned off, and the absence of another protein that regulates cell growth. The newest evidence also shows that Cd arsenite can lower the viability of yeast cells by blocking the unfolding of new proteins. This has a big impact on many diseases, such as neurological diseases, age-related diseases, and neurodegenerative disorders like Parkinson’s and Alzheimer’s (31). Furthermore, Cd exhibition is considered a possible hazardous ingredient for bone density, even though crucial levels of exposure and accurate processes remain unclear. Also, it has been established that prenatal exposure to Cd influences a child’s brain and kidneys (48). Disruption of the blood-testis barrier disrupts testosterone synthesis, which in turn leads to diminished germ cell adhesion, germ cell death, decreased sperm count, and either subfertility or infertility (49–51).

**Figure 1:**
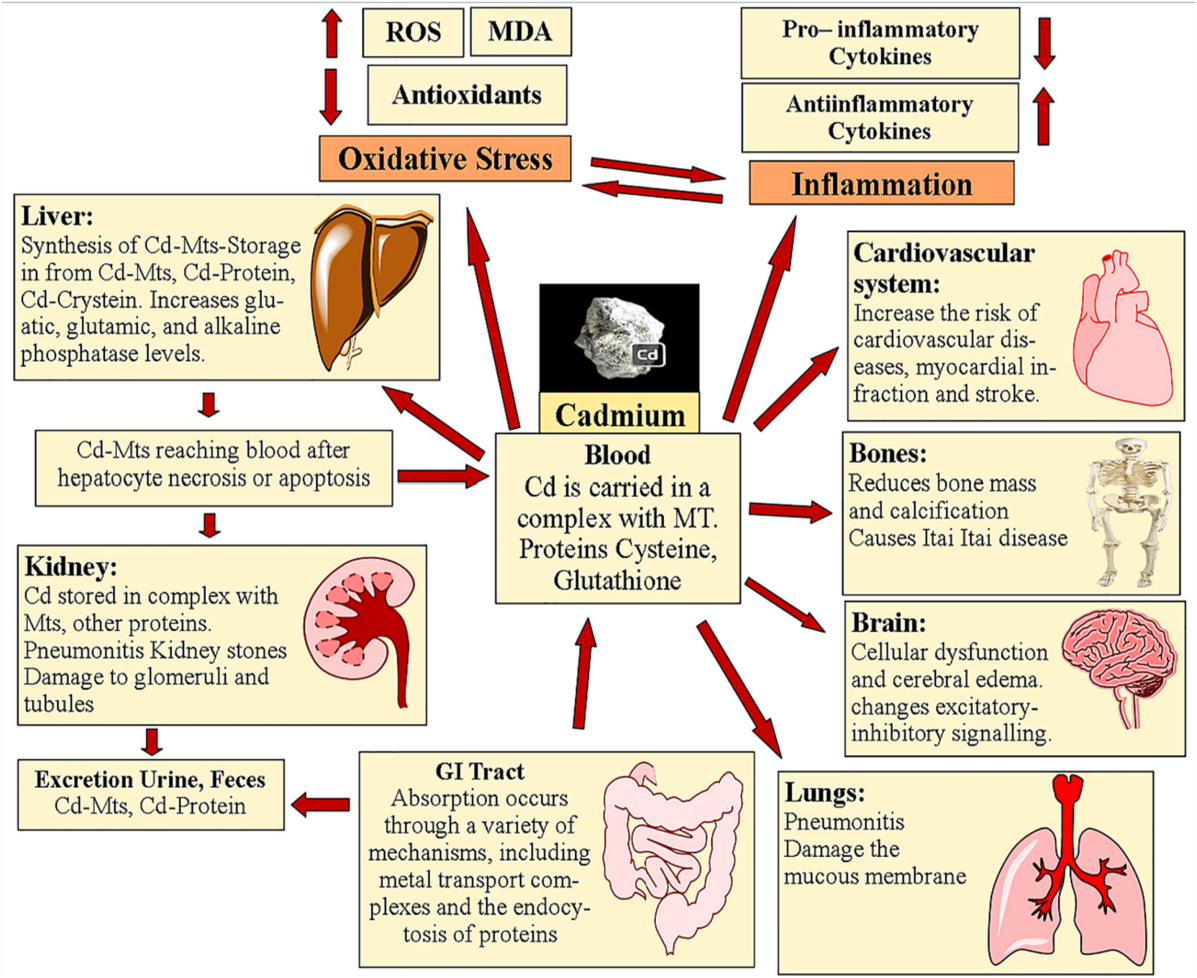
Toxicity of Cd and its dispersion inside the body.

Some antioxidant enzymes, like CAT, manganese SOD, glutathione, GPx, glutathione reductase, and copper-zinc superoxide dismutase, cannot work as well when Cd is present (17, 27, 52). MT is a protein that can scavenge free radicals and concentrates the mineral Zn. In cells, the presence of MTs confers resistance to the harmful effects of Cd, but the inability to produce MTs makes cells more susceptible to the metal’s effects (53). In cases of Cd-induced toxicity, the expression of MTs is what determines whether apoptosis or necrosis will take place (54). The consequences of Cd hitting mitochondria include oxidative stress, the production of ROS, the start of apoptosis, the mutation of MTs-DNA, the modification of gene expression, the inhibition of respiratory chain complexes, the reduction of ATP synthesis, and the modification of endo-mitochondrial conductivity. Such mitochondrial effects allow the evaluation of a wide range of human illnesses (55, 56). Cd′s toxic effects and accumulation locations in the body are illustrated in Figure 1.

This review mentioned that cadmium exposure is a global concern, with millions of people being exposed to this toxic heavy metal annually (17). The long-term accumulation of cadmium in the body and its association with various health issues, such as organ toxicity and degenerative disorders, highlights the importance of understanding cadmium toxicity and finding effective remediation strategies.

## 3 Therapeutic efficacy of medicinal plants in mitigating cadmium-induced toxicity

Several cancer statistics reports, including Cancer Statistics (American Cancer Society 2012), showed an estimated 1.64 million new cases of cancer detected and an alarming number of approximately 577,000 deaths caused by cancer in the United States of America (57), while India estimated 14.61427 million new cases of cancer detected alone in 2022 (58). It has been demonstrated that cancer is a disease that can be prevented to some extent, and it is thought that dietary components may be able to modulate cancer risk. This is despite the fact that cancer is one of the key causes of death across the globe.

Natural resources, such as herbal plants, comprise an extensive array of phytochemicals that hold promise as conventional remedies for chronic and infectious ailments. In contrast to synthetic agents, these resources are regarded as secure and efficacious alternatives that exhibit a reduced incidence of adverse effects (59). It has been estimated that appropriate dietary modifications can prevent 30–50 percent of all cancers. Dietary patterns, foods, nutrients, and other nutritional components are intimately related to the possibility of several kinds of cancer (60). According to several epidemiological and animal studies, a diet rich in fruits and vegetables can lower your chances of developing many illnesses (60). This indicates that specific dietary elements may be useful in preventing cancer (61). Some nutrients, dietary supplements, and naturally occurring substances found in nutraceuticals (62, 63) may help fight some diseases by reducing oxidative stress. These substances can be found in herbal medicines, vitamins, amino acids, and processed foods and drinks like cereals, drinks, gums, and turmeric. Flavonoids and terpenoid indole alkaloids are the primary nutraceutical components of plants (TIAs) (64). There was a favorable effect on Alzheimer’s disease symptoms in the research involving plant extracts such as *C. roseus*, *Zizyphus jujube,* and *Lavandula officinalis* (64, 65).

### 3.1 Significance of using medicinal plants and herbs as alternative medicines

Many of the phytochemicals included in foods like fruits, seeds, herbs, plants, and grains have been linked to lowering cancer hazards. Plants produce polyphenols, which are secondary metabolites used in defense against microbial, insect, and herbivore attacks. Dietary nutrients derived from plants have been linked to acting as active ingredients in some herbal and traditional medications (29). Researchers have found that polyphenols can help fight different types of cancer, protect the heart, brain, and joints, lower blood sugar, stop cancer from spreading, and protect the kidneys and liver (29, 66, 67). Bioactive compounds are classified as alkaloids, flavonoids, and polyphenols. These are composed of heterocyclic nitrogen, which is generally basic, which makes them extremely therapeutic. Indole alkaloids and polyphenols are widely spread and abundantly present in different plant families (68). A lot of different phenolic compounds, flavonoids, and secondary metabolites can be found in plants that have aromatic rings with at least one hydroxyl group (69). Herbal remedies typically contain phytochemical compounds. It has been observed that flavonoids and several alkaloids can act as antioxidants, anticancer agents, antibacterial agents, anti-inflammatory agents, and interesting applicants for pharmaceutical and medical applications (70, 71). The Indian subcontinent is home to the ancestors of some of the plants and herbs. These plants and herbs contain apparent probable therapeutic characteristics that are acknowledged in Ayurvedic and Unani, the traditional medical systems of India and worldwide, respectively. In a variety of research models, it was shown that a significant number of traditional medicines possessed a feature that defended them against the poisonous effects of heavy metals.

Ginger, *Sesamum indicum*, has natural antioxidants that greatly reduce lipid peroxidation and heavy metal toxicity (72, 73). It has been shown that medicines like *Albizia amara, Allium sativum, Ocimum sanctum, Datura stramonium, Teucrium polium L., Crataeva nurvala Buch-Ham, Urtica dioica L.,* and *Dracocephalum molsdavica L*. can fight lipid peroxidation and a number of illnesses (74–76). Vitamin E can also shield the cell membrane from the deteriorating results of oxidative stress and lipid peroxidation (77). Studies done on living things have shown that alpha-tocopherol’s antioxidant properties can stop damage caused by Cd and lower oxidative stress (78). Polyphenol curcumin, found in turmeric, is known to prevent lipid peroxidation (43). Coenzyme Q10 (CoQ10), unlike vitamins, is generated from phenylalanine and mevalonic acid endogenously in the body. An instrumental function helps in avoiding lipid peroxidation from commencing and harming biomolecules (79). It helps to lessen the lipid peroxidation that Cd toxicity causes (80). N-acetyl cysteine, an amino acid found in nature, blocks lipid peroxidation by an autocatalytic mechanism (81). Quercetin is a flavonoid that can be found in onions and tomatoes. It has been shown to protect rats’ brains from Cd-induced neurotoxicity by blocking the ion of lipid peroxide (82, 83).

The antioxidant activities of apple polyphenol extract have been shown to have a protective impact against oxidative stress in the liver (28). Several of the damaging effects of Cd on organs, including the liver and kidney, can be prevented or slowed by taking vitamins A, C, E, and selenium (23). Herbal medicines like *A. sativum L, S. marianum, Z. officinale, W. somnifera, O. sanctum, E. officinalis, R. officinalis, P. ginseng, and C. roseus* (17, 84) reduced the amount of Cd that built up in the liver and kidneys. Antioxidant properties can be found in alpha-lipoic acid (ALA). Reportedly, ALA causes regulating effects on Cd toxicity and slows down the detrimental effects of Cd, which results in significant decreases in Cd residues in the liver and kidneys (17, 85). Resveratrol and antioxidant quercetin reduced Cd-induced oxidative damage to the kidneys, oxidative stress, and liver injury in rats (86).

### 3.2 Overview of *Catharanthus roseus* (L.)

The Indian subcontinent is home to a diverse range of medicinal plants, many of which have not been researched to their full potential. The evergreen herbaceous plant *C. roseus* is also known as rose *Catharanthus.* In common parlance, periwinkle is referred to as “Nayantara” or “Sadabhar.” The name “*Catharanthus roseus*” comes from the Greek language and literally translates to “perfect flower.” The word “roseus” can also indicate red, rose, or rosy. It has been discovered that this plant is critically endangered in its natural habitat, and the principal reason for their dwindling numbers is the destruction of their natural habitat by agricultural practices that involve slashing and burning (9, 87).

This plant has a long history of medicinal applications, and it has been used to cure a wide variety of illnesses. For ages, people in Europe used it as a home cure to treat diabetes (88). Wasp stings were alleviated using the juice extracted from the leaves in India. Poultices used to staunch bleeding were traditionally made in Hawaii by boiling the plant. In China, people used it as a treatment for coughs as well as for astringent and diuretic uses (9). In Central and South America, it was used as a home medicine for the common cold to relieve symptoms of inflammation. Oral administration of a humid extract of dehydrated leaves is used to treat menorrhagia and diabetes in Australia, and oral administration of an extract of root bark is used to treat fever (9, 88).

### 3.3 Phytochemical constituents found in *Catharanthus roseus*

Several studies have shown that terpenoids and phenylpropanoids, along with sugars, flavonoids, saponins, and even alkaloids, make up the main parts of *C. roseus* (17). Alkaloids are the *C. roseus* chemical ingredient with the greatest potential for biological activity. In terms of chemicals, the plant has more than 200 different types of alkaloids, such as catharanthine, actineoplastidemeric, reserpine, raubasine, ajmalicine, Vinceine, vinneamine, raubasine, and more (89, 90). These alkaloids are used in a variety of industries, including pharma-ceuticals, agro-chemicals, flavoring and perfume, components, artificial additives, etc.

The roots, leaves, and basal stem of this plant contain various alkaloids, including the well-known chemically used anti-carcinogenic alkaloids vinblastine, vindoline, vindolidine, vindolicine, vindolinine, vindogentianine, and vincristine (9). The flower of the *C. roseus* plant contains an anthocyanin pigment known as rosindin (91). These compound groups’ antioxidant properties are well documented and also aid in compensating for the oxidative damage that heavy metals cause (17).

Important by-products of this plant include anhydro-vinblastine, vindoline, catharanthine, ajmalicine, and serpentine. *Catharanthus roseus* has a lot of bisindole alkaloids, which are about 40 different constituents, and a lot of them have a vindoline and catharanthine moiety. A few alkaloids found in *C. roseus* are used in the pharmaceutical industry (92, 93). Vincristine, also known as leucocristine and Oncovin R, was first developed in 1963. It is an oxidized version of the drug vinblastine. Even though these compounds can be extracted from the leaves of the plant, the herb itself excludes large amounts of them while it is still living. Vindoline and catharanthine are two of the precursor alkaloids that are found in plants (in the waxy coating of the leaves). It is impossible to synthesize specific compounds for use in cancer treatment without first combining these precursors (9, 92, 94).

**Figure 2:**
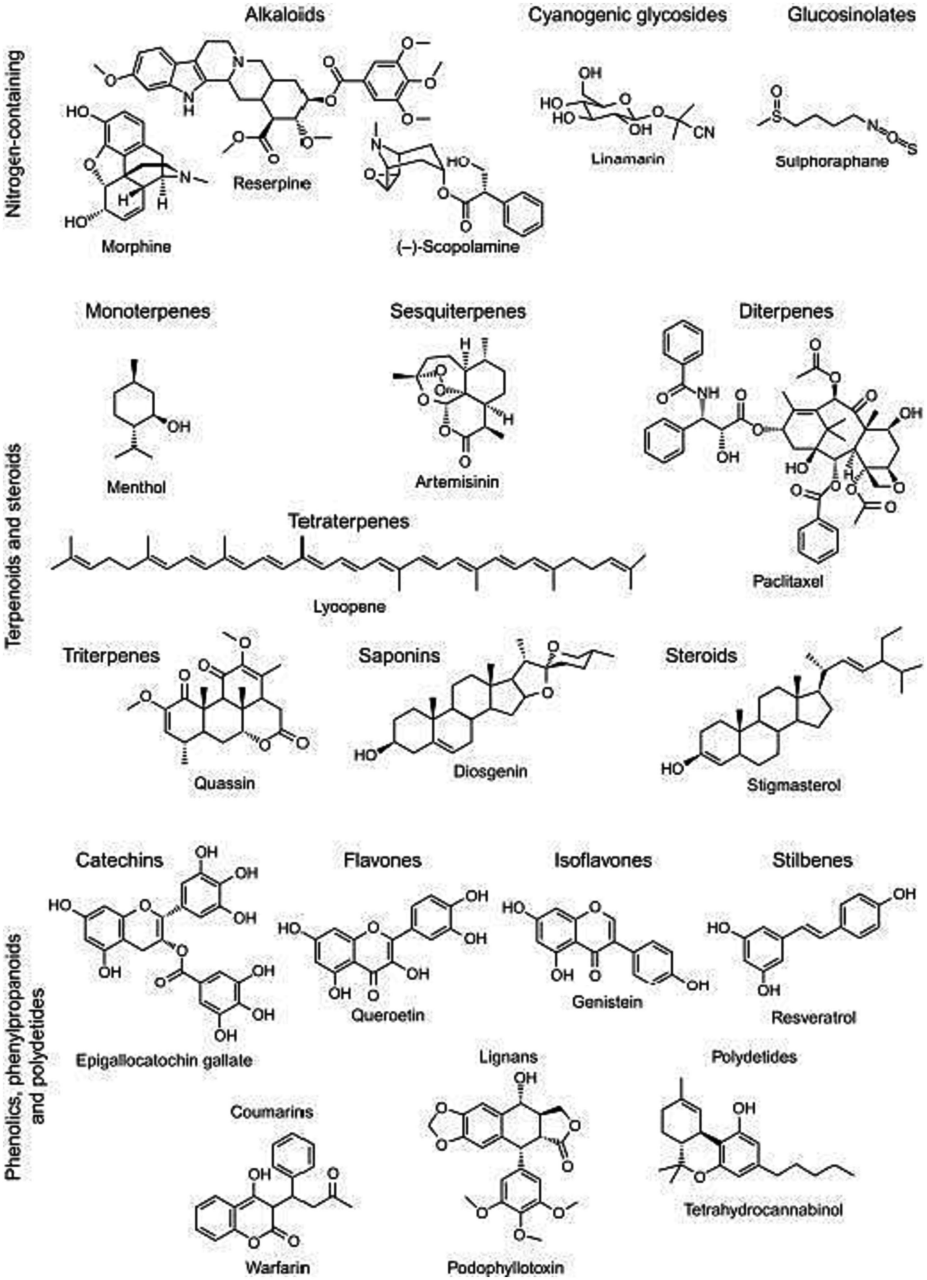
Main phytoconstituent of *Catharanthus roseus* extract source (94, 97).

Terpenoids such as monoterpenes and carotenoids, along with polyphenols like quercetin and other flavonoids, encompass essential phytochemicals known for their wide array of antioxidant effects (95). Phenolic compounds are, found in various plants, share a common feature of having one or more hydroxyl substituents attached to aromatic or benzene rings, and they can be grouped into phenolic acids, flavonoids, stilbenoids, and lignans. These compounds exhibit a wide array of therapeutic properties, including anticancer, anti-inflammatory, and antioxidant effects, with their antioxidant potential dependent on the number and position of hydroxyl groups in their structures (96). Terpenoids, like menthol (Figure 2), play a vital role in maintaining fruit quality by preserving levels of sugars, organic acids, anthocyanins, and antioxidant capacity in fruits like strawberries and tomatoes. Carotenoids, used widely in the food industry, are divided into hydrocarbon carotenoids (carotenes) and oxygenated derivatives (xanthophylls) and serve as both dyes and secondary antioxidants, effectively scavenging reactive oxygen species due to their conjugated double bonds, thus helping prevent oxidative damage (94, 98). Quercetin having higher no of hydroxyl group in both ring a and b tends to have a higher efficacy in protecting lipid membrane in plants. This property is attributed to its higher antioxidants’ potential depending on the str activity study (96).

### 3.4 Therapeutic properties of *Catharanthus roseus*

*Catharanthus roseus*, recognized as a significant pharmaceutical plant source among over 21,000 plants and herbs, has been historically employed to treat various health issues, ranging from hyperglycemia and sore mouth to leukemia, cancer, and diabetes (10). People have reported using this plant for diverse ailments, including memory loss, high blood pressure, wasp sting pain, gum bleeding, mouth ulcers, and nosebleeds. Since ancient times, *C. roseus* has been utilized in diabetes treatment and hypertension due to the belief that it either stimulates insulin synthesis or enhances the body’s ability to utilize sugar (99). Studies on animal have demonstrated that the ethanolic extract, derived from leaves and flowers, can lower blood glucose levels and inhibit the formation of new metarterioles, crucial for preventing tumor growth (93).

*Catharanthus roseus* exhibits a broad range of pharmaceutical effects, with phytoconstituents used to address various ailments such as loose bowels, Alzheimer’s disease, asthma, throat infections, digestive issues, toothache, skin conditions, and more (88, 92, 93). The herb serves as a vital source of therapeutic compounds directly related to herbalism, toxicology, pharmacognosy, and organic chemotherapy drugs and therapies. However, several studies suggested that the aqueous or alcoholic dried leaf extract might not have any negative effects on myeloid tissue, immune system, GI tract, kidney, or liver (17, 100). In addition to its usefulness as an ornament, it possesses a variety of pharmaceutical qualities, such as antibacterial, antioxidant, antidiarrheal, anti-inflammatory, antioxidant, and anti-carcinogenic activities (17, 101, 102). Alkaloids, the primary phytochemical element of *C. roseus*, are utilized in a wide variety of therapeutic applications. Alkaloids are a type of plant molecule that has a pungent taste and is primarily composed of nitrogen. Research has shown that many alkaloids have qualities that alleviate pain. *C. roseus* contains cancer-fighting alkaloids, including vinblastine and vincristine, which are important in the prevention of the disease (93, 100, 101).

Furthermore, it should be noted that three recently discovered dimeric indole alkaloids namely 17-deacetoxycyclovinblastine, 14′,15′-didehydrocyclovinblastine, and 17-deacetoxyvinamidine along with five established compounds (vinamidine, leurosine, catharine, cycloleurosine, and leurosidine) exhibited *in vitro* viability inhibition against the human breast cancer cell line MDA-MB-231 with an inhibitory concentration ranging from 0.73 to 10.67 μM (44). Significantly, cathachunine, a novel bisindole alkaloid from *C. roseus*, decreased the activity of leukemia cells while having considerably lesser cytotoxicity against normal human endothelium cells, showing that its activity was inhibited specifically toward leukemia cells (93).

In addition to research on the anticancer effects of individual alkaloids from *C. roseus*, research was also done on the whole crude extract on different types of cancer cells. Researchers recently discovered that a product from the roots and stems of *C. roseus* was very good at killing several cancer cell lines in the lab (93). Fernández-Pérez et al. (103) also discovered that the strong anticancer effect of the indole alkaloid-rich extract from *C. roseus* cell cultures was not caused by a single compound but by the action of several bioactive compounds working together. Together, these results show that the bioactive parts of *C. roseus* work well together to kill cancer cells. This effect has been seen in other plant products (104, 105) and is being thought of as a way to treat cancer (106). This phenomenon can be explained by compounds that have little to no activity, helping the main active component reach its target through mechanisms of action that complement each other, reverse resistance, improve bioavailability, reduce metabolism and excretion, and modulate side effects (93). This study underscores the potential of *C. roseus* extract as a natural remedy to counteract the harmful effects of cadmium and promote overall organ health.

### 3.5 The significance ameliorative efficacy of *Catharanthus roseus* extract on Cd-induced toxicity

Several reports have observed that plant-based medications and their ingredients are fast being employed to treat a variety of harmful disorders in humans, either explicitly or implicitly (107). Herbal medicines, vegetables, and fruits that are high in polyphenols, such as terpene indole alkaloids, flavonoids, vitamins, and minerals, can fight oxidative stress in a number of ways. They may even be able to help with the bad effects of too many toxic metals. Moreover, the accessibility and characteristics of these plant-based elements play a significant role in indicating preservation. In this investigation, we attempted to demonstrate that exogenously administered *C. roseus* extracts can provide protection against metal cytotoxicity. According to Hashim et al. (17), *C. roseus* extract contains bioactive substances, including polyphenols acting as antioxidants. The protective effect of *C. roseus* extract on the kidney and liver from Cd toxicity is crucial, as oxidative cell damage can lead to various health issues, including kidney and liver diseases. *Catharanthus roseus* extract contains bioactive substances, including polyphenols acting as antioxidants. Moreover, significantly reduced glomerulosclerosis, vascular endothelium, and tubular serious injuries, with “considerable preservation of serum waste substances, DNA breakage, and normalization of protein profiles, hematological parameters, MDA concentration, and antioxidant enzyme levels” (6, 17). This made chemotherapeutic medicine less harmful, increased people’s lifespans, and made it easier for better health care systems to develop.

## 4 Conclusion

In conclusion, the study into how *C. roseus* extract protects against cadmium-induced toxicity in animal models has led to useful findings that could have therapeutic implications. Cd, a highly hazardous heavy metal, poses severe health risks to both humans and animals due to its pervasive environmental contamination. This review indicates that the administration of *C. roseus* extract exhibits a substantial capacity to mitigate the adverse consequences of Cd exposure. Important considerations when utilizing *C. roseus* extract to lessen the negative effects of Cd include its impact on the economy and the environment. The economic implications include the cost of production, extraction, and implementation, as well as the potential impact on market prices for other remediation strategies. This analysis is crucial in determining the feasibility and sustainability of using *C. roseus* extract as a remediation strategy for Cd toxicity. Ongoing research efforts aim to address these concerns and identify ways to improve the economic and ecological efficiency of implementing *C. roseus* extract.

*Catharanthus roseus* extract possesses potent antioxidant properties that are crucial for squelching free radicals and reducing the oxidative stress that Cd causes. The extract also has chelating properties that allow it to bind to Cd ions and make it easier for the body to get rid of them. These mechanisms collectively contribute to the alleviation of Cd-induced damage to various organs and physiological systems, encompassing the liver, kidneys, and reproductive system. Additionally, the study underscores that the extract has the potential to rectify histopathological abnormalities and restore normal biochemical parameters perturbed by cadmium toxicity. By bringing these parameters back to their normal levels, the *C. roseus* extract shows that it is effective at protecting against Cd-induced toxicity, which highlights its potential as a natural medicine. The implications of these research findings extend to the sphere of both human and animal health, considering the persistent global concern regarding cadmium exposure. The extract of *C. roseus* shows promise as a way to prevent and treat Cd toxicity, which would lower the health risks that come with it. Nevertheless, it is imperative to conduct further investigations, including clinical trials, to ascertain the safety, efficacy, and optimal dosage of the extract in human subjects.

## Author contributions

MH: Conceptualization, Investigation, Writing – original draft, Writing – review & editing, Formal analysis, Resources, Visualization. HA: Supervision, Visualization, Writing – review & editing. BT: Resources, Supervision, Writing – review & editing. SR: Writing – review & editing. PB: Writing – review & editing. RS: Writing – review & editing. MK: Writing – review & editing.
